# A series of quaternary ammonium salt antibacterial agents synthesized and prepared for constructing and screening antibacterial coatings with biosafety on polypropylene

**DOI:** 10.3389/fmicb.2026.1718331

**Published:** 2026-01-29

**Authors:** Leixiang Wang, Shukai Nan, Qiaozhi Wang, Yinuo Xu, Meng Cui, Fenglai Wang, Yaxuan Liu, Guige Hou, Zhonghao Liu, Wenjuan Zhou, Yu-Qing Zhao

**Affiliations:** 1 School of Pharmacy, Key Laboratory of Medical Antibacterial Materials of Shandong Province, Binzhou Medical University, Yantai, China; 2 Nanjing Medical University, Nanjing, China; 3 Institute of Stomatology, Binzhou Medical University, Yantai, China

**Keywords:** antibacterial, anti-inflammatory, biocompatibility, polypropylene, quaternary ammonium salt

## Abstract

Diseases caused by bacteria have become the world’s largest threat, and the treatment of bacterial infections urgently needs to be addressed. However, the abuse of antibiotics leads to superbugs, making bacterial infections more difficult to resolve. Therefore, there is an urgent need to develop new antibacterial agents. In this study, three antibacterial agents were synthesized. *In vitro* antibacterial experiments demonstrated that the antibacterial agent quaternized polyethyleneimine (QPEI) possessed favorable antibacterial activity and exhibited good antibacterial performance against a diverse array of bacteria and fungus, such as *Escherichia coli*, *Staphylococcus aureus*, *Pseudomonas aeruginosa*, and *Candida albicans*. QPEI-C6 has an inhibitory concentration of 8 μg/mL against *Staphylococcus aureus*, 128 μg/mL against *Escherichia coli*, 16 μg/mL against *Candida albicans*, and 32 μg/mL against *Pseudomonas aeruginosa*. Furthermore, through antibacterial and cell biocompatibility experiments, it was shown that QPEI-C6 had good biocompatibility and excellent antibacterial performance within the concentration range of 8–128 μg/mL. The antibacterial agent QPEI-C6 combined with the natural polyphenol tannic acid (TA) was subsequently employed to modify the surface of polypropylene (PP) material, leading to outstanding bactericidal, anti-inflammatory, and antioxidant efficacies. The hemolysis rate of the final material group was 3.4%, and the *in vitro* cell survival rate was as high as 110%. The antibacterial rate against *S. aureus* reaches 99%. On the surface of the modified material, excessive reactive oxygen species (ROS) could be effectively eliminated, and the generation of oxidative stress was significantly mitigated. Anti-inflammatory experiments indicated that the coating substantially reduced the expression levels of TNF-*α* and IL-6 while promoting the release of IL-10. In this work, the cationic antibacterial agent QPEI was successfully synthesized, and the PP material was surface modified. A suite of materials with excellent antibacterial, antioxidant, and biocompatibility properties, which have positive and significant implications in the biomedical field, are presented in this work.

## Graphical abstract

**Figure:**
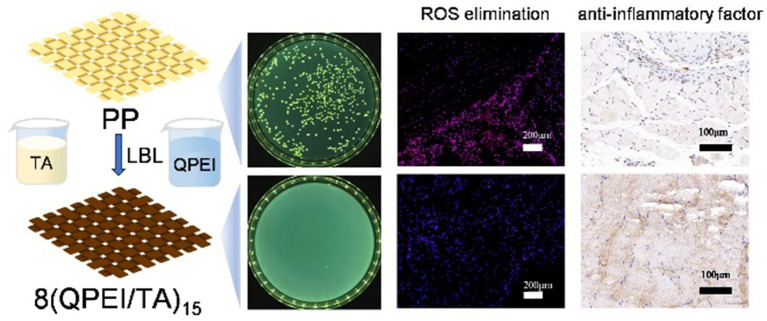

## 1 Introduction

Bacterial infection is the most serious health problem in the world, seriously restricting the improvement of human living standards (Lan et al., 2024). When the human body is injured, bacteria accumulate and grow on the surface of the wound (Tang et al., 2023). Biomedical materials (Le Visage and Chew, 2023) are new high-technology materials used for diagnosing, treating, repairing, or replacing damaged tissues or organs or enhancing their functions in living organisms (Wu et al., 2024). There are two main types of bacterial infections that affect biomedical materials during use: first, when biomedical materials are used (Ding et al., 2024), these materials may become infected by small amounts of bacteria present in human wounds, skin, or mucous membranes (Zhou et al., 2021); second, bacteria will adhere to the surface of medical materials, aggregate, and grow, forming relatively strong biofilms, which could cause bacterial infections (Wang et al., 2022). In response to these issues, antibiotics are currently widely used for treatment (Horne et al., 2024). However, antibiotics have significant side effects, and bacteria can develop resistance, which could prolong the treatment time and increase the patient’s pain while creating an economic burden (Li et al., 2021).

Therefore, to avoid bacterial infections and reduce side effects on the human body, researchers have proposed various methods of surface modification of biomedical materials (Jiang et al., 2023). Contact sterilization is achieved mainly by modifying the surface of materials with quaternary ammonium salts (Jitsuhiro et al., 2024), antimicrobial peptides (Mohanraj et al., 2018), etc., by physical and chemical methods. When bacteria come into contact with the modified surface, they are adsorbed, and their structure is destroyed to achieve antibacterial effects. An alternative approach is a slow-release surface coating that uses antibiotics or metal ions (Weng et al., 2023) as antibacterial agents, which are released to kill bacteria when stimulated, degraded, or swollen.

Since the 1950s, polypropylene (PP) patches have been recognized as the gold standard for hernia repair surgery (Wang et al., 2020), with advantages such as high tensile strength, stable properties, easy cutting, and strong and elastic support for hernia defect sites (Katkhouda et al., 2020). However, polypropylene materials exhibit biological inertness and are nonabsorbable in the body, which may lead to some complications. Dopamine (DA) can form a PDA coating on the surface of inert materials by oxidative self-polymerization under alkaline conditions (Liu et al., 2021; Awonusi et al., 2022). This coating can react with compounds containing amino, thiol and other functional groups through the structure of catechol to form stable chemical bonds (Zamboni et al., 2023), with good biocompatibility and other advantages (Davidsen et al., 2021).

Polymer antibacterial agents present many advantages, such as excellent stability, long-lasting antibacterial activity, and low residual toxicity (Haktaniyan and Bradley, 2022). Importantly, polymeric antibacterial agents have strong antibacterial efficacy because of the high local density of their active groups (Guo et al., 2014). Notably, cationic antibacterial agents containing quaternary ammonium and phosphate groups exhibit excellent antibacterial activity because of their high charge density, and they possess good processing properties (Zhang et al., 2025). Therefore, many researchers have committed to developing polymer antibacterial agents with different chemical structures to meet the needs of different fields (Bureš, 2019; Fedorowicz and Sączewski, 2024). Most of the antibacterial cationic polymers reported in the literature are derived from quaternary ammonium compounds, polyethyleneimine (PEI) derivatives, and chitosan derivatives (Xu et al., 2018).

Quaternization is a chemical reaction process that produces quaternary ammonium salt compounds (Zhou et al., 2022). Quaternary ammonium salt compounds have shown wide application value because of their unique chemical structure and they can be used as surfactants, antibacterial agents, and antistatic agents. They can also be used in textile and papermaking processes. Quaternization, a commonly used modification method, is often used to improve the solubility and antibacterial ability of natural polysaccharides (Luo et al., 2025).

Polyethylene imine (PEI) is a water-soluble polymer produced through the polymerization of ethylene imine (Karatepe and Ozdemir, 2020). It is a partially branched polymer containing primary, secondary, and tertiary amines. Polyethyleneimine molecular chains contain many amino N atoms, making them cationic agents with strong antibacterial activity (Li B. Y. et al., 2025). However, they have a certain degree of cytotoxicity (Beyth et al., 2008; Xu et al., 2024), and their cationic nature is strongly affected by pH. PEI can be quaternized to prepare the cationic polyelectrolyte quaternized polyethyleneimine (QPEI). QPEI utilizes its unique physical membrane breaking mechanism - cation electrostatic adsorption and hydrophobic chain insertion - to synergistically disrupt bacterial cell membranes, avoiding the defect of traditional antibiotics that are prone to developing resistance due to targeting specific metabolic pathways (Lan et al., 2019). It not only exhibits broad-spectrum and efficient killing ability against Gram positive bacteria, Gram negative bacteria, multidrug-resistant bacteria (such as MRSA), and fungi, but also effectively penetrates and removes stubborn biofilms. The clearance rate of related materials (such as bone regeneration scaffolds) on biofilms can reach over 99.9%. At present, QPEI has been successfully applied to implant device coatings, anti infective tissue engineering scaffolds, and synergistic therapy systems, demonstrating great potential in addressing drug-resistant bacterial infections and chronic biofilm infections (Tong et al., 2020).

Tannic acid (TA) (Lin et al., 2020) is a naturally occurring weakly acidic polyphenolic compound that appears as a yellow or brownish yellow powder (He et al., 2021). TA has a catechol structure and can interact with other substances through various hydrogen bonding and electrostatic interactions (Maia et al., 2025). Its polyphenolic hydroxyl structure endows it with excellent antioxidant properties, which can clear free radicals from a wound site and protect the wound from inflammatory cell attacks (Utatsu et al., 2023).

Layer-by-layer self-assembly (LBL) technology (An et al., 2018) is a simple and multifunctional surface modification method that was rapidly developed in the 1990s (Bai et al., 2022). LBL is a cyclic process (Richardson et al., 2016; Zhang et al., 2022) that involves alternating cyclic deposition on a material substrate (Li Q. et al., 2025). The proposed method has notable advantages, characterized by its straightforward operation and extensive applicability for the fabrication of antibacterial surfaces on various substrates (Wang et al., 2023).

On the basis of the above information, a stable surface coating of biomedical materials was constructed by systematically exploring the antibacterial properties of branched PEI and QPEI. First, we studied the antibacterial activity and cytotoxicity of PEI. On this basis, different branched QPEI were synthesized with 1-bromohexane, 1-bromooctane, and 1-bromodecane alkylating reagents. The antibacterial activity and cytotoxicity of PEI and different branched QPEI molecules were systematically studied, ultimately leading to the selection of suitable cationic antibacterial agents, which provides a theoretical basis for the further development and wide use of PEI and QPEI in applications requiring antibacterial and antibiofilm properties.

Following systematic evaluation of the antibacterial activity and cytotoxicity of these compounds, an appropriate branched QPEI was identified as a cationic antibacterial agent for subsequent surface functionalization. The selected branched QPEI and TA were grafted to the surface of the material through layer-by-layer self-assembly. On the basis of our previous work, QPEI was used as a cationic polyelectrolyte, TA was used as an anionic polyelectrolyte, electrostatic adsorption could be generated between QPEI and TA, and the phenolic structure on the TA structure could produce hydrogen bonding with the amino group on QPEI, thus forming a stable coating (Li et al., 2021).

**SCHEME 1:**
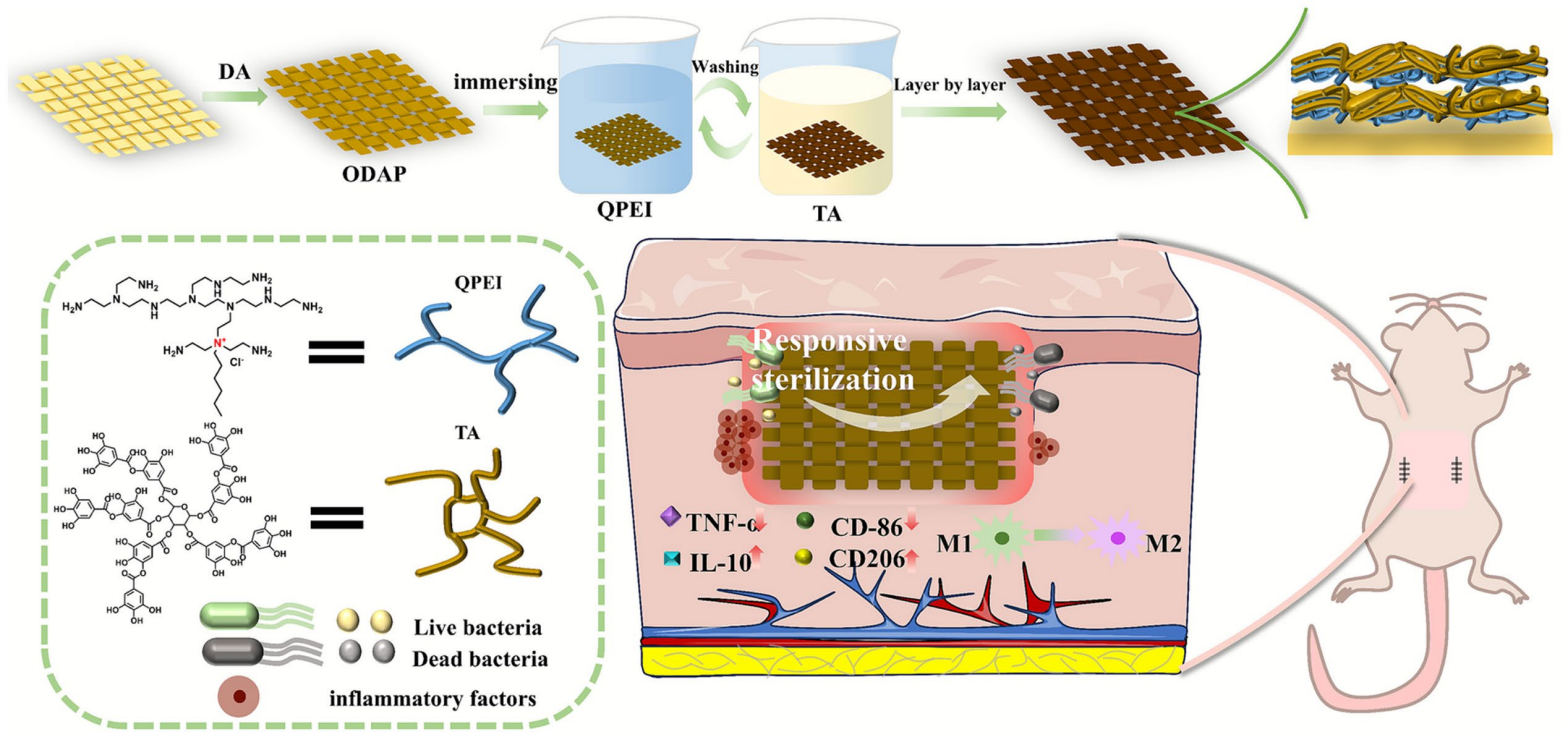
Schematic illustrations of the antibacterial and anti-inflammatory properties of QPEI/TA.

In this work, we further investigated the appropriate surface bactericidal concentration of QPEI and a surface modified with different layers of coatings of (QPEI/TA)_n_ at different concentrations, ultimately selecting the most suitable coating for the study of biomedical materials (Scheme 1).

## 2 Experimental section

### 2.1 Materials

Ethylene imine polymer, 1-bromohexane, 1-bromooctane, 1-bromodecane, n-hexane, n-heptane, n-decane, K₂S₂O₈, tetrahydrofuran, tris(hydroxymethyl) aminomethane, tannic acid, potassium persulfate, 2,2-diphenyl-1-picrylhydrazyl and 2,2′-azino-bis(3-ethylbenzothiazoline-6-sulfonic acid) diammonium salts were purchased from Macklin (Shanghai, China). Dopamine hydrochloride was purchased from Alfa Aesar (China), and ethyl alcohol was purchased from Yuandong (Yantai, China). Dulbecco’s modified Eagle’s medium (DMEM) was purchased from Cytiva (USA). The cell fixative was purchased from Solarbio (Beijing, China). Yeast extract, tryptone, and agar were purchased from Thermo Fisher. A cell proliferation toxicity detection kit (CCK-8) was purchased from Labgic (Beijing, China, C2015S). *S. aureus*, *E. coli*, *P. aeruginosa*, and *C. albicans* (SHBCC, ATCC 25923, ATCC 25922, ATCC 27853, ATCC 14028). ELISA Kit IL-6 (4A BIOTECH CME0006). Mouse IL-10 ELISA KIT (Shanghai Zhen Ke Biological Technology Co. Ltd. ZK4375). Mouse TNF-*α* ELISA KIT (Shanghai Zhen Ke Biological Technology Co. Ltd. ZK4558). Mouse fbroblast L929 cells and murine macrophage RAW264.7 cells were obtained from Beijing Beina Chuanglian Biotechnology Research Institute. BALB/c mice (Shandong Pengyue Laboratory Animal Technology CO., Ltd.,).

### 2.2 Synthesis of QPEI-Cn

Three 2 g portions of polyethyleneimine (PEI) were weighed and dissolved in 8 mL of tetrahydrofuran (THF). A total of 1.8 g of 1-bromohexane, 2.1 g of 1-bromooctane, or 2.4 g of 1-bromodecane was weighed and dissolved in 8 mL of THF (Equivalent to a molar ratio of 10:1 between halogenated hydrocarbons and polyethyleneimine). The PEI solution was slowly added dropwise to the bromohexane, bromooctane, and bromodecane solutions, which were subsequently stirred and allowed to react at 50 °C for 12 h. After the reaction was complete, the supernatant was collected and precipitated with 10 times the amount of N-hexane under stirring conditions. The precipitate was collected by centrifugation (1,500 rpm, 10 min), and the product was a yellow viscous substance. Subsequently, vacuum drying was performed to obtain QPEI-C6 featuring branches of different lengths, along with those of QPEI-C8 and QPEI-C10 (Li et al., 2024).

### 2.3 *In vitro* antibacterial activity of QPEI-Cn

In a 5 mL centrifuge tube, 6.144 mg of the cationic antibacterial agent polyethyleneimine (PEI) and three kinds of quaternized polyethyleneimines (QPEI-C6, QPEI-C8, and QPEI-C10) with different modifications were weighed and irradiated with UV light for 12 h. On an ultraclean workbench, each of the UV-irradiated samples was dissolved in 3 mL of culture solution. The formulation concentration of the antibacterial agent was 2048 μg/mL. Gradient dilution was performed with the culture mixture to generate PEI and QPEI solutions with concentrations of 2048, 1,024, 512, 256, 128, 64, 32, 16 and 8 μg/mL. In a 96-well plate, 100 μL of bacterial and fungal suspension (*S. aureus*, *E. coli*, *P. aeruginosa*, or *C. albicans*) at a concentration of 10^8^ CFU/mL was added to each well. Subsequently, 100 μL of different concentrations of antibacterial agents were added successively. At this time, the concentrations of antibacterial agents in the wells were 1,024, 512, 256, 128, 64, 32, 16, 8 and 4 μg/mL. There were three parallel groups for each concentration. The control group consisted of the same volume of culture medium and bacterial suspension. The samples were incubated at 37 °C for 12 h. Following the culture period, 40 μL aliquots of coculture solution were collected from each well, subjected to 100-fold dilution, and subsequently plated. After the corresponding marks were made, the petri dishes were transferred to a biochemical incubator and maintained for 12 h, after which bacterial growth was observed and recorded (Gao et al., 2008).

### 2.4 *In vitro* biocompatibility test

Cytotoxicity experiments were carried out on the PEI, QPEI-C6, QPEI-C8 and QPEI-C10 samples. The four samples were prediluted to 8, 16, 32, 64, 128, 256, 512, 1,024, and 2048 μg/mL and then sterilized.

L929 cells at the logarithmic growth phase were harvested through trypsin digestion and centrifugation, resuspended, and adjusted to a density of 4 × 10^8^ cells/mL per well. Following cultivation to 80% confluency, the medium was replaced with the prepared sample solution at the specified concentration. Three parallel samples were set in each group, and a group of cells without samples was set as the control group. Then, the samples were placed in a cell incubator and cultured at 37 °C with 5% CO_2_ for 24 h. The culture medium was replaced with 10% CCK-8 prepared in serum-free medium, followed by incubation in the cell incubator for 40 min. Then, 100 μL samples were pipetted into a 96-well plate, and absorbance measurements were conducted at 450 nm using a microplate reader (Gibney et al., 2012). The formula for calculating cell viability is as follows:


$$\text{Cell activity}\;(\%)=[\frac{OD_{\text{test}}}{OD_{\text{cont}}}]\times 100\%$$ (1)

Where OD_test_ is the absorbance of different concentrations in the experimental group at 450 nm and OD_cont_ is the absorbance of the bacterial suspension in the control group at 450 nm.

### 2.5 Preparation of (QPEI/TA)_n_

The polypropylene (PP) material was cut into two squares (1.5 × 1.5 cm and 2.0 × 2.0 cm) with scissors, and then the dust and other impurities attached to the surface of the material were washed away with detergent. The material was placed in a beaker, absolute ethanol or distilled water was added, ultrasonic cleaning was performed three times, and finally, the material was dried in a 50 °C oven. Tris (hydroxymethyl) aminomethane (0.121 g) was weighed and dissolved in 100 mL of water to obtain a Tris buffered solution. Dopamine hydrochloride (0.2 g) was weighed and dissolved in 100 mL of Tris buffered solution to obtain a 2 mg/mL dopamine solution. The cleaned PP material was placed in dopamine solution and reacted at 37 °C for 4 h. After the reaction was completed, the sample was rinsed with distilled water three times to remove nonadherent dopamine and then placed in an oven to dry.

QPEI (0.14 g, 0.35 g and 0.56 g) was weighed and dissolved in 70 mL of distilled water through ultrasonication to obtain 2 mg/mL, 5 mg/mL, and 8 mg/mL QPEI cationic solutions, respectively. Similarly, 0.14 g, 0.35 g and 0.56 g of TA were weighed and added to 70 mL of distilled water for ultrasonic dissolution to obtain 2 mg/mL, 5 mg/mL and 8 mg/mL tannic acid TA anionic solutions, respectively. The pH was subsequently adjusted to 7.4 with 1 M HCI and 1 M NaOH.

The number of prepared layers was controlled to 10, and the times were 10, 15 and 20 min. QPEI and TA at the same concentrations were used for layer-by-layer self-assembly. DAPP was placed in a 2 mg/mL QPEI cationic solution, stirred, and then soaked for 10 min, followed by cleaning with deionized water; then, the material was placed in a 2 mg/mL TA anionic solution, stirred and soaked for 10 min. This process was a cycle; that is, one layer was assembled, and the procedure was repeated 10 times to obtain 10 layers. Following the same process, the preparation of the other groups of materials was completed.

There were nine different groups: 2(QPEI/TA)_10_, 5(QPEI/TA)_10_, 8(QPEI/TA)_10_, 2(QPEI/TA)_15_, 5(QPEI/TA)_15_, 8(QPEI/TA)_15_, 2(QPEI/TA)_20_, 5(QPEI/TA)_20_, and 8(QPEI/TA)_20_. Following the completion of material preparation, photographs were taken, and experimental records were documented. DAPP was modified with QPEI and TA at a concentration of 8 mg/mL for 15 min according to the above steps. Following the assembly of the final layer in the QPEI solution, the sample was washed with deionized water. A portion of the sample was removed and not immersed in TA solution, resulting in the 8(QPEI/TA)_15_-Q group, whereas the remaining portion was further assembled in TA solution to generate the 8(QPEI/TA)_15_-T group.

### 2.6 *In vitro* antibacterial activity

The PP, DAPP, 2(QPEI/TA)_10_, 5(QPEI/TA)_10_, 8(QPEI/TA)_10_, 2(QPEI/TA)_15_, 5(QPEI/TA)_15_, 8(QPEI/TA)_15_, 2(QPEI/TA)_20_, 5(QPEI/TA)_20_, and 8(QPEI/TA)_20_ were cut into 0.6 cm diameter discs with a punch and then placed in 96-well plates. Bacterial cultures at the logarithmic growth phase were diluted to a concentration of 1 × 10⁶ CFU/mL, followed by pipetting and the addition of 50 μL aliquots onto each sample surface, ensuring complete infiltration. The cells were incubated in a shaker at 150 rpm for 5 h at 37 °C. Following the coculture period, each sample’s bacterial solution was diluted with sterile PBS to an appropriate concentration, and 100 μL aliquots were transferred for plate coating. The coated plates were inverted and incubated in a 37 °C bacterial incubator for 18–24 h (Gao et al., 2008). The bacterial colonies were subsequently photographed and enumerated using a colony counter, and the bacteriostatic rate was subsequently calculated.


$$\text{Bacteriostatic rate}=[\frac{OD_{\text{cont}}-OD_{\text{test}}}{OD_{\text{cont}}}]\times 100\%$$ (2)

Where Q_cont_ is the number of viable bacteria in the control group on the agar plate and Q_test_ represents the number of viable bacteria in the experimental group on the agar plate.

### 2.7 Free radical scavenging performance

A total of 7.3 mg of ABTS was weighed and dissolved in a 5 mL centrifuge tube containing 2 mL of distilled water, and 1.3 mg of K₂S₂O₈ was weighed and dissolved in another 5 mL centrifuge tube containing 2 mL of distilled water. The two solutions were mixed and reacted for 12 h at room temperature in the dark. After the reaction, 1 mL of the mixture was removed, and 33 mL of distilled water was added to dilute it to obtain the ABTS working solution. The 1.5 × 1.5 cm material was put into 15 mL centrifuge tubes, 5 mL of ABTS working solution was added to each tube, and it was wrapped with aluminium foil to avoid light, with the ABTS working solution set as the control group. At 0 min, 15 min, 30 min, 1 h, 2 h, 4 h, 6 h, 10 h, and 24 h, 100 μL of solution was pipetted and transferred to a 96-well plate, with three parallel samples in each group (Li et al., 2024). The absorbance at 734 nm was measured with a microplate reader, and the clearance rate was calculated with the following formula:


$$A\mathrm{BTS}\;\text{scavenging}=[\frac{OD_{\text{abts}}-OD_{\text{sample}}}{OD_{\text{abts}}}]\times 100\%$$ (3)

DPPH (2 mg) was weighed and transferred to a 100 mL light-protected sample bottle wrapped with aluminium foil, followed by the addition of 50 mL absolute ethanol. Materials measuring 1.5 × 1.5 cm were placed into 15 mL centrifuge tubes, and 5 mL of DPPH solution was added to each tube. The tubes were wrapped in aluminium foil to prevent light exposure, with a separate DPPH solution serving as the control group. At specified time points (0 min, 15 min, 30 min, 1 h, 2 h, 4 h, 6 h, 10 h, and 24 h), 100 μL aliquots were transferred to a 96-well plate, with triplicate samples for each group. The absorbance was measured at 517 nm using a microplate reader, and the clearance rate was calculated with the following formula:


$$\text{DPPH scavenging}=[\frac{OD_{\text{dpph}}-OD_{\text{sample}}}{OD_{\text{dpph}}}]\times 100\%$$ (4)

### 2.8 *In vivo* antibacterial experiment

Eight-week-old BALB/c mice were acclimatized for 3 days prior to experimentation. Following anesthesia, a 1 cm incision was made on both sides of the mouse spine to separate the skin and muscle tissue. A 0.6 cm diameter material was implanted bilaterally, with polypropylene (PP) placed on the left side and 8(QPEI/TA)_15_ on the right. Each sample was inoculated with 5 μL 10^8^ CFU/mL of a *Staphylococcus aureus* suspension before suturing the incision to establish a bacterial infection model. On postoperative days 3 and 7, mice were euthanized, and the implanted discs were explanted and transferred into 1 mL of phosphate-buffered saline (PBS) in centrifuge tubes. The samples were vortexed for 30 s, serially diluted (100 ×), and plated onto solid LB agar to quantify bacterial viability. Additionally, muscle tissue sections were subjected to hematoxylin and eosin (H&E) (Shanghai Jingke Chemical Technology Co., Ltd. 71014460) staining to assess inflammatory cell infiltration (Li Q. et al., 2025).

### 2.9 *In vivo* anti-inflammatory, antioxidant and immune regulatory efficacy of 8(QPEI/TA)_15_

On postoperative days 3 and 7, the muscle tissue surrounding the implant was stained with dihydroethidine (DHE) (Servicebio G1045). DHE readily diffuses across cell membranes and is oxidized by intracellular reactive oxygen species (ROS) to form ethidium, which intercalates into DNA and emits red fluorescence. The intensity of red fluorescence was quantified to determine intracellular ROS levels. Additionally, the expression of inflammatory cytokines TNF-*α* (BOSTER BA0131) and IL-10 (BOSTER BA1201-1) in the peri-implant muscle tissue was evaluated. TNF-α, a pro-inflammatory cytokine, mediates inflammatory responses, whereas IL-10, an anti-inflammatory factor, suppresses inflammation and counteracts inflammatory mediators. The staining intensity of positively labeled cells in immunohistochemical sections correlated with the degree of inflammation. To further verify the material’s ability to modulate the local inflammatory microenvironment, immunofluorescence staining was performed on muscle tissue samples collected on days 3 and 7 to assess the expression of M1 macrophage marker CD86 (BOSTER BM4121) and M2 macrophage marker CD206 (abcam ab64693). Since inflammation suppression can mitigate excessive tissue fibrosis, collagen deposition around the implant was evaluated using Masson’s trichrome (Beijing Solarbio Science & Technology Co., Ltd. G1340) staining (Li Q. et al., 2025).

### 2.10 Anesthesia and euthanasia method

During the induction period, BALB/c mice were placed in the induction chamber and treated with a carrier gas flow rate of 1–2 L/min and a concentration of 3–5% isoflurane for 2 min. After experiencing gait instability and immobility in the prone position, they were removed; During the maintenance period, use a mask with a carrier gas flow rate of 0.8–1.2 L/min and an isoflurane concentration of 2.0–2.5%, while observing the skin color to ensure that the hind limbs do not retract; Close the volatilization tank during the awakening period, continue supplying oxygen for 5–10 min, and keep warm with a 37 °C heating pad (Li et al., 2024).

BALB/c mice were in a closed anesthesia chamber, adjust the concentration of isoflurane to 5–8%, and maintain a carrier gas flow rate of 1–2 L/min. Expose for 5–10 min until breathing and heartbeat stop.

### 2.11 Statistical analysis

All data in this study were analyzed via GraphPad Prism software, and t tests were used to compare the differences between two groups. When two or more groups were compared, analysis of variance (ANOVA) was used. At least 3 samples per group were used.

## 3 Results and discussion

### 3.1 Preliminary material synthesis and characterization

The synthesis of QPEI was characterized by Fourier transform infrared (FT-IR) and NMR spectroscopies. QPEI has an absorption peak at approximately 2,924 cm^−1^, indicating the presence of CH_2_- groups (Supplementary Figure S1a). Further verification of the synthesis of QPEI via ^1^H NMR revealed (Supplementary Figure S1b) that the 2.4–0.4 ppm peak was associated with the chemical shift of N-CH_2_, the 1.29 ppm peak was associated with the chemical shift of CH_2_-, and the 0.87 ppm peak was associated with the chemical shift of CH_3_, which is consistent with the structure of QPEI, confirming the successful preparation of QPEI.

### 3.2 Broad-spectrum antibacterial activity of QPEI

**Figure 1:**
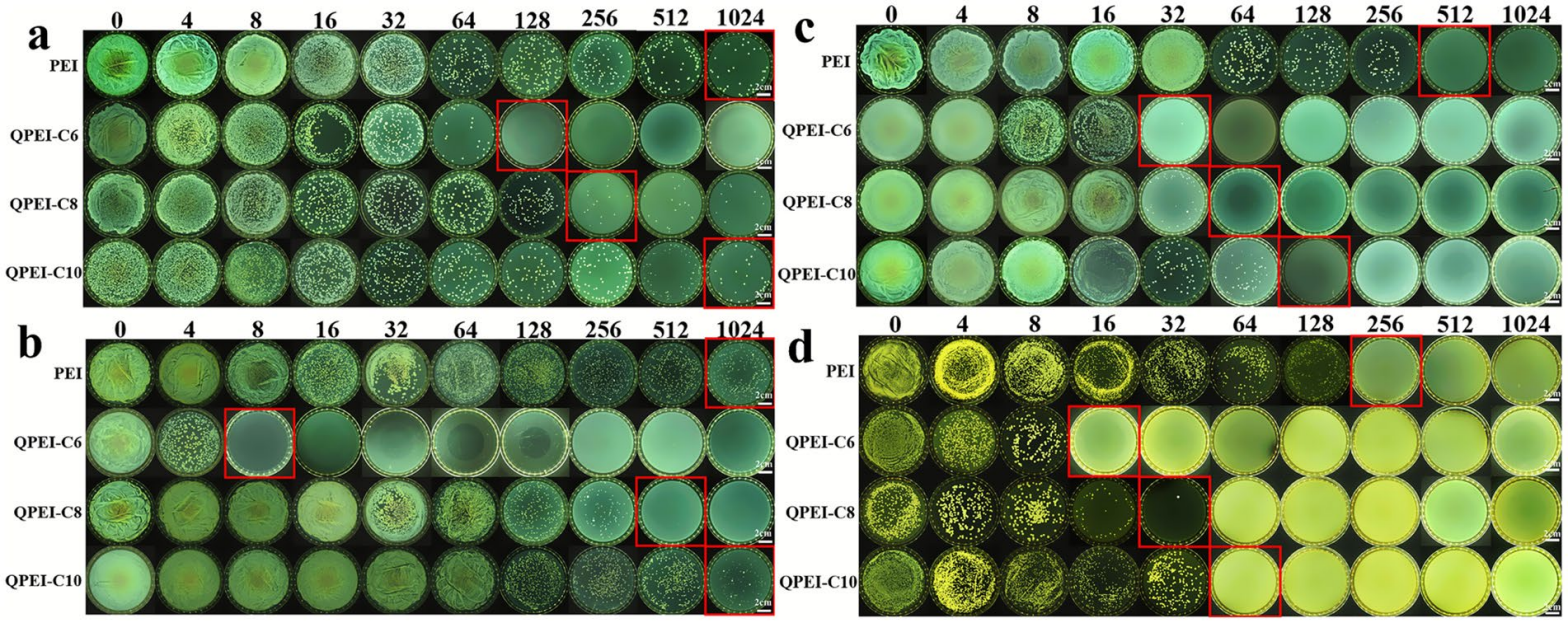
Images of surviving bacterial colonies on agar plates after co-culture of *E. coli* **(a)**, *S. aureus* **(b)**, *P. aeruginosa* **(c)**, and *C. albicans* **(d)** with different concentrations of polyethyleneimine (PEI) and its three quaternized derivatives (QPEI-C6, QPEI-C8, QPEI-C10) (μg/mL).

In this study, the antibacterial efficacies of PEI, QPEI-C6, QPEI-C8, and QPEI-C10 were determined via the plate count method. First, antibacterial experiments were conducted on two common types of bacteria. When the concentration of PEI reached 1,024 μg/mL, there was no significant damage to *E. coli*, indicating that the antibacterial effect of PEI was relatively low. In contrast, when the suspension of *E. coli* was coincubated with QPEI-C6, the number of colonies on the agar plate decreased with increasing QPEI-C6 concentration. In particular, when the QPEI-C6 concentration was 128 μg/mL, no colonies were observed on the agar plate, indicating that almost all the bacteria were killed at this concentration (Figure 1a). Similarly, when the concentration of PEI reached 1,024 μg/mL, there was still no significant damage to *S. aureus*, indicating that the antibacterial efficacy of PEI is relatively low. However, QPEI-C6, QPEI-C8, and QPEI-C10 showed strong inhibitory effects against *S. aureus* at concentrations of 8, 512, and 1,024 μg/mL, respectively (Figure 1b).

Subsequently, the antibacterial activity against *P. aeruginosa* was tested. *P. aeruginosa* is an opportunistic pathogen and is one of the main pathogens causing hospital-acquired infections. As shown in Figure 1c, PEI and QPEI-C10 exhibited significant antibacterial properties at 64 μg/mL, while QPEI-C6 and QPEI-C8 showed excellent antibacterial properties at 32 μg/mL against *P. aeruginosa*.

Finally, the antifungal activity against *C. albicans* was determined. *C. albicans* is a common fungus that can cause acute and chronic infections in the skin, mouth, and other areas. As shown in Figure 1d, PEI had good antifungal performance at a concentration of 256 μg/mL, QPEI-C6 had significant antifungal properties at 16 μg/mL, while QPEI-C8 and QPEI-C10 exhibited excellent antifungal properties at 32 μg/mL and 64 μg/mL, respectively (Table 1).

**Table 1:** Polyethyleneimine (PEI) and its three quaternized derivatives (QPEI-C6, QPEI-C8, QPEI-C10) exhibit minimum inhibitory concentrations (MIC) against *E. coli*, *S. aureus*, *P. aeruginosa*, and *C. albicans in vitro* (μg/mL).

Microbial species	PEI (μg/mL)	QPEI-C6 (μg/mL)	QPEI-C8 (μg/mL)	QPEI-C10 (μg/mL)
*E. coli*	1,024	128	256	1,024
*S. aureus*	1,024	8	512	1,024
*P. aeruginosa*	512	32	64	128
*C. albicans*	256	16	32	664

In comparison, QPEI-C6 displayed potent antimicrobial activity against gram-positive (*S. aureus*) and gram-negative (*E. coli*, *P. aeruginosa*) bacteria, as well as against the fungus *C. albicans,* at reduced concentrations, whereas PEI, QPEI-C8, and QPEI-C10 showed minimal efficacy against these pathogens even at relatively high concentrations.

### 3.3 Biocompatibility assessment of QPEI

The biocompatibility of the material was evaluated through cytotoxicity assays to assess its potential adverse effects on cellular viability and function. The order of cytotoxicity from highest to lowest was as follows: QPEI-C6 > QPEI-C8 > QPEI-C10 > PEI (Supplementary Figure S2a). The cytotoxicity of QPEI decreases as the length of the halogenated hydrocarbon undergoing quaternization increases after the quaternization reaction. Through cell toxicity experiments, it was verified that QPEI-C6 at 128 μg/mL, which has excellent bactericidal effects, still had good cell compatibility, with a cell viability above 80%. However, PEI, QPEI-C8, and QPEI-C10 no longer had good cell compatibility at bactericidal concentrations. On the basis of the above experimental results, QPEI-C6 was selected as the antimicrobial material for subsequent experiments.

### 3.4 Synthesis and characterization of functionalized surface layers on materials

The functional layer on the material surface was constructed via layer-by-layer (LBL) assembly technology using quaternized polyethyleneimine (QPEI) and tannic acid (TA). Polyethyleneimine (PEI) was quaternized to yield QPEI, which carries a substantial number of positively charged groups, whereas TA contains abundant negatively charged hydroxyl groups. Consequently, these two compounds form stable coatings through electrostatic interactions between opposite charges and intermolecular hydrogen bonding. Prior to LBL assembly, polypropylene (PP) was coated with polydopamine (PDA) and designated DAPP. Subsequently, DAPP was functionalized with QPEI/TA multilayers, and the resulting materials were labeled (QPEI/TA)_n_, where “n” represents the deposition time.

**Figure 2:**
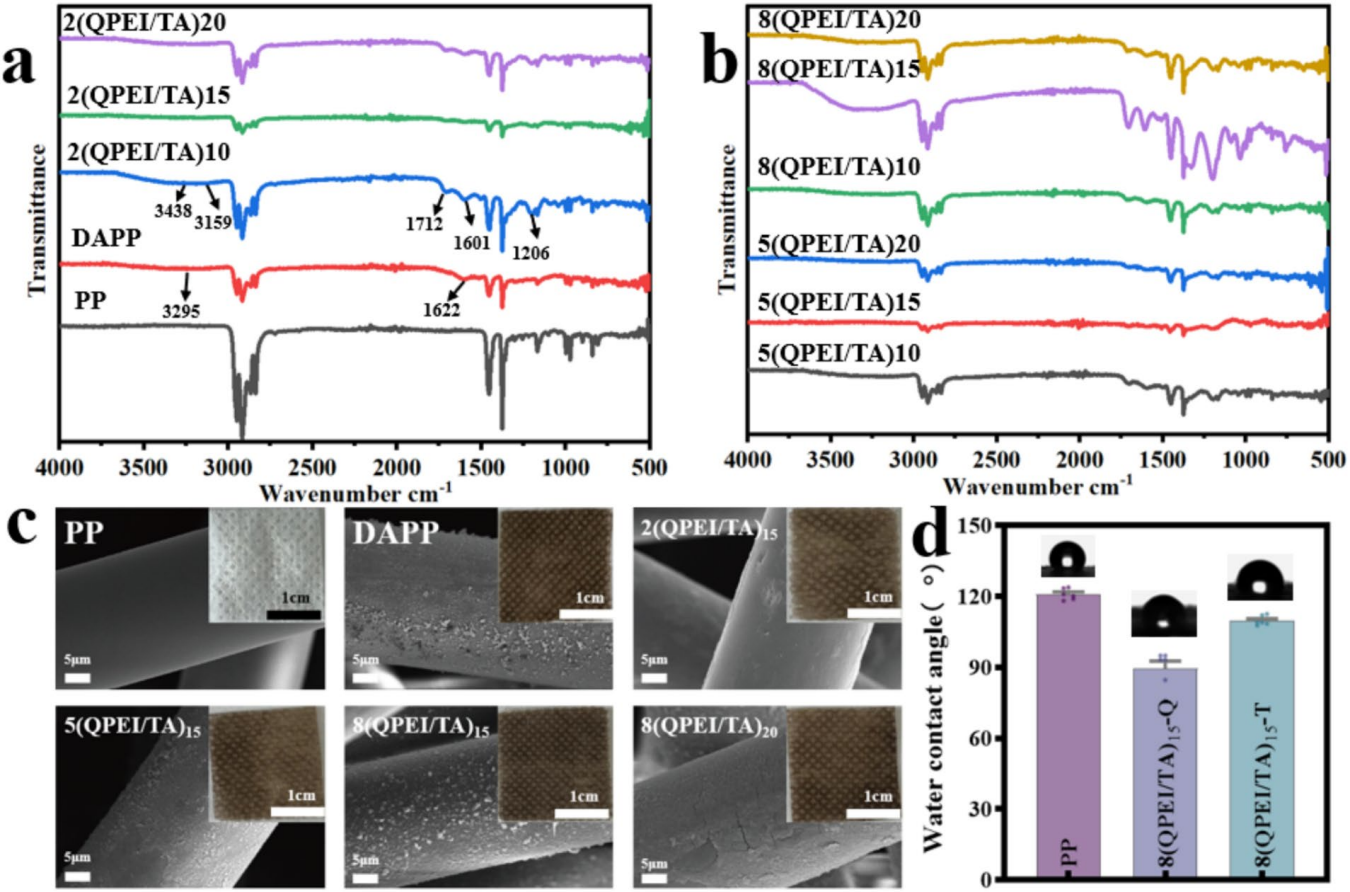
Infrared spectral scanning images of the materials before **(a)** and after **(b)** layer-by-layer self-assembly. **(c)** Images of PP, DAPP, and materials modified with the QPEI/TA surface coating and SEM images of each sample. **(d)** Water contact angle tests on the material’s surfaces.

The FT-IR results of each sample are shown in Figures 2a,b. The peaks at 3295 cm^−1^ and 1,622 cm^−1^ observed in DAPP were caused by the stretching and vibrational deformation of the amino group of dopamine. The 3,438 cm^−1^ peak in the sample modified with QPEI/TA was attributed to intermolecular hydrogen bonding generated by the phenolic hydroxyl group in tannic acid; the 3,159 cm^−1^ peak was attributed to the amino group; the 1,712 cm^−1^ peak and 1,206 cm^−1^ peak were caused by C=O and -O- in TA, respectively; while the 1,601 cm^−1^ peak was offset to a certain extent from the 1,622 cm^−1^ peak in DAPP due to hydrogen bonding between QPEI and TA.

The appearance and morphology of the prepared material are shown in Figure 2c. The raw material polypropylene (PP) appears white. The sheetlike and smooth surface of the dopamine-functionalized DAPP and QPEI/TA assembly materials darkened in color, and the surface became rough. The microscopic morphologies of the PP, DAPP, 2(QPEI/TA)_15_, 5(QPEI/TA)_15_, and 8(QPEI/TA)_15_ samples were observed using SEM (Figure 2c). The figure shows that the surface of the PP was smooth and that there were granular substances on the surface of DAPP, 2(QPEI/TA)_15_, 5(QPEI/TA)_15_, and 8(QPEI/TA)_15_. The surface of the samples had coatings, indicating that the QPEI/TA was successfully grafted onto the surface of the material.

The water contact angles of PP, 8(QPEI/TA)_15_-Q, and 8(QPEI/TA)_15_-T were measured using a contact angle goniometer to evaluate the changes in hydrophobicity before and after surface modification, as illustrated in Figure 2d. The water contact angle of the PP was approximately 120°, the water contact angle of the 8(QPEI/TA)_15_-Q material was approximately 90°, and the water contact angle of the 8(QPEI/TA)_15_-T material was approximately 110°. The hydrophobic PP material exhibited varying degrees of hydrophilicity after surface modification.

### 3.5 Biocompatibility

**Figure 3:**
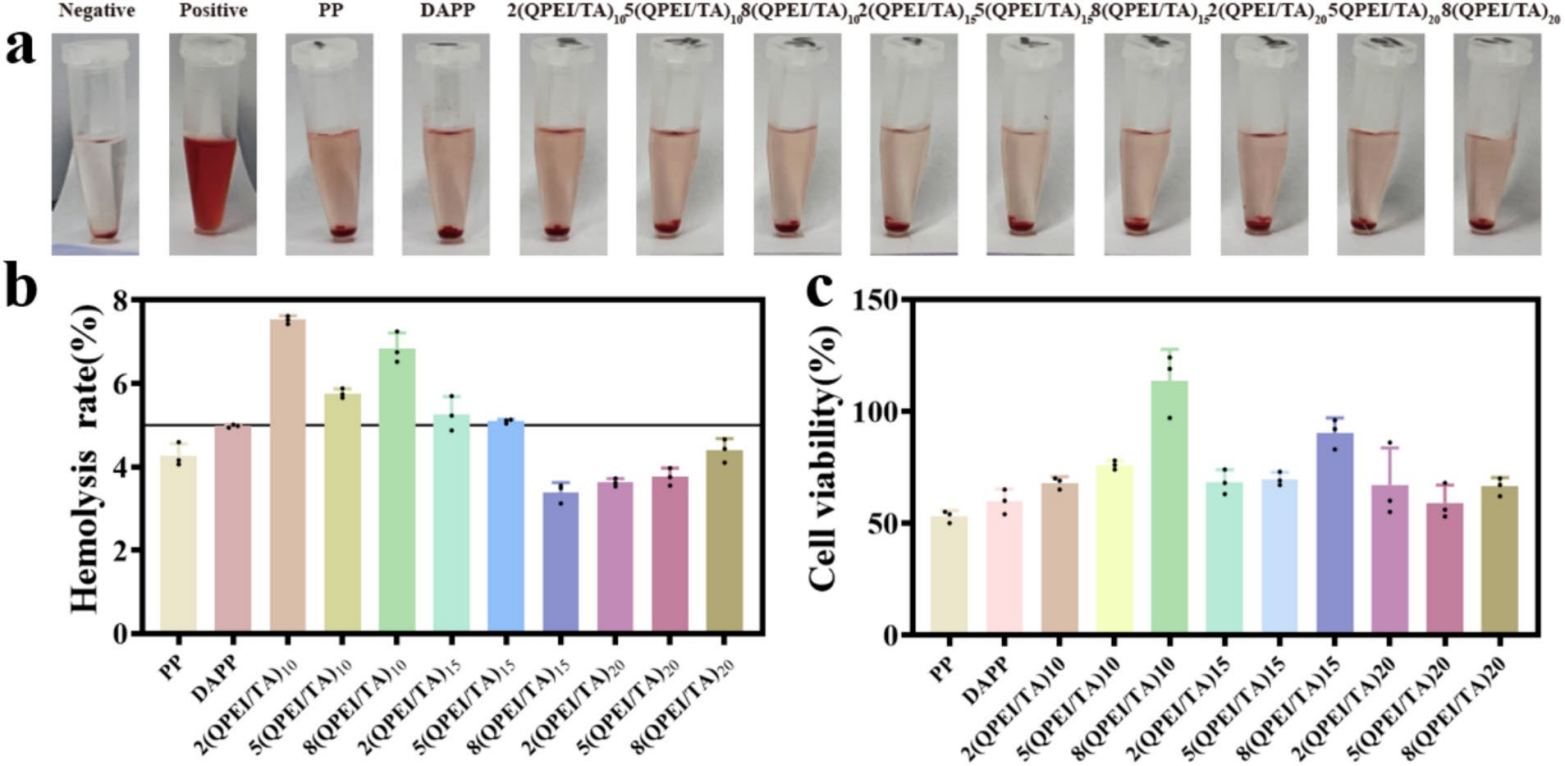
**(a)** Hemolysis diagrams of different samples. **(b)** Calculate the hemolysis rate of different samples after instrument measurement. **(c)** Measure the survival rate of L929 cells after co culturing with different materials.

The results of the hemolysis tests are shown in Figure 3a, and the hemolysis rate was calculated and is shown in Figure 3b. If the hemolysis rate was greater than 5%, the material was considered to cause an unacceptable rate of hemolysis. Therefore, the hemolysis rates of groups PP, 8(QPEI/TA)_15_, 2(QPEI/TA)_20_, 5(QPEI/TA)_20_, and 8(QPEI/TA)_20_ meet the requirements, with 8(QPEI/TA)_15_ having the lowest hemolysis rate among all the groups, approximately 3.4%.

Cell toxicity testing is an important indicator for evaluating the biocompatibility of medical materials. A CCK-8 reagent kit was used. The cell survival rates were calculated, and the materials’ cytotoxicity was analyzed (Figure 3c). The survival rate of cells treated with 8(QPEI/TA)_10_ was approximately 110%, indicating that the cytotoxicity of this group was extremely low and did not affect the normal growth of the cells. The second highest survival rate occurred with 8(QPEI/TA)_15_, with a cell survival rate of approximately 90% and low cytotoxicity.

The hemolysis and cytotoxicity assays revealed that 8(QPEI/TA)_10_ presented the highest cell survival rate but its hemolysis rate exceeded the acceptable threshold. In contrast, the other materials presented lower hemolysis rates but significantly greater cytotoxicity. Among all the groups, 8(QPEI/TA)_15_ achieved a balance, showing both a high cell survival rate and a low hemolysis rate that met the required standards, indicating superior biocompatibility. Consequently, the functional layer of this group was selected for further experimental validation in subsequent studies.

### 3.6 Antioxidant properties analysis

**Figure 4:**
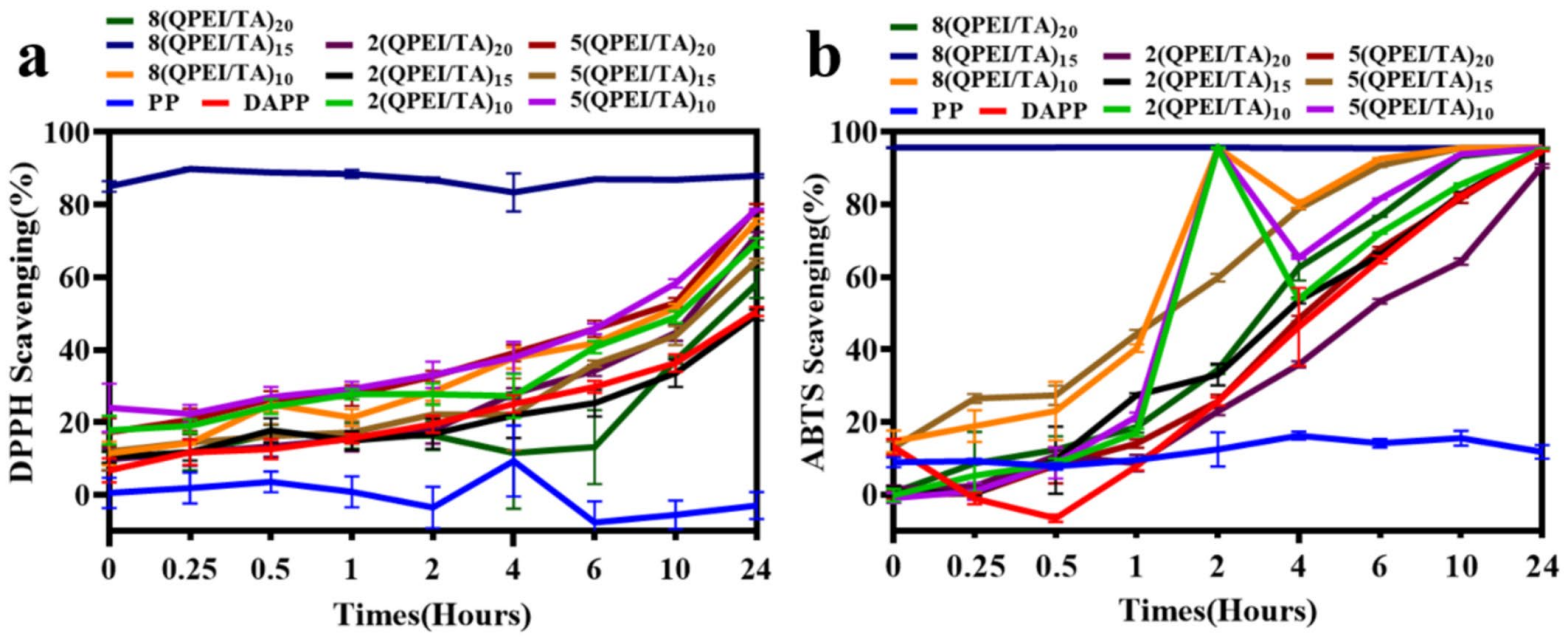
**(a)** Line graph of clearance rates of each group of samples after co-incubation with ABTS and **(b)** line graph of clearance rates of each group of samples after co-incubation with DPPH.

The experimental results showed that when tested with both the ABTS method (Figure 4a) and the DPPH method (Figure 4b), 8(QPEI/TA)_15_ had the highest free radical scavenging rates, the fastest scavenging time and the best scavenging effect compared with the other materials. In the ABTS experiment, the free radical scavenging rate of 8(QPEI/TA)_15_ immediately reached more than 95%; the free radical scavenging rates of the other groups gradually increased to approximately 90% over time, but the rate of increase was slow. In the DPPH experiments, the free radical scavenging rate of 8(QPEI/TA)_15_ reached 87%. The free radical scavenging rates of the other groups of materials slowly increased to approximately 75%, and their free radical scavenging ability was worse than that of 8(QPEI/TA)_15_. The PP group showed little change overall and did not show the ability to scavenge free radicals. In the DPPH experiment, the clearance of the other groups increased slowly over time.

### 3.7 Antibacterial properties analysis

**Figure 5:**
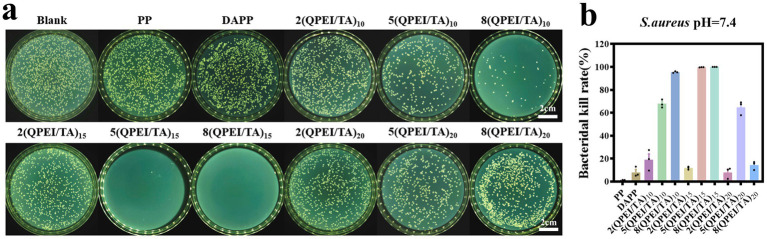
**(a)** Agar images of viable bacteria co-cultured with different groups of materials and **(b)** antibacterial rates of different groups of materials against *S. aureus.*

*Staphylococcus aureus* was selected as the representative bacterium for antibacterial experiments. The samples of PP, DAPP, 2(QPEI/TA)_10_, 5(QPEI/TA)_10_, 8(QPEI/TA)_10_, 2(QPEI/TA)_15_, 5(QPEI/TA)_15_, 8(QPEI/TA)_15_, 2(QPEI/TA)_20_, 5(QPEI/TA)_20_, and 8(QPEI/TA)_20_ were subjected to plate antibacterial tests at pH 7.4. The surviving bacterial colonies were imaged and quantified using a colony counter (Figure 5a), and the inhibition rates were subsequently calculated and graphically represented (Figure 5b). The antibacterial rate of 5(QPEI/TA)_15_ was greater than 99%, while that of 8(QPEI/TA)_15_ reached 100%, demonstrating excellent antibacterial ability_._

### 3.8 *In vivo* antibacterial assessment

**Figure 6:**
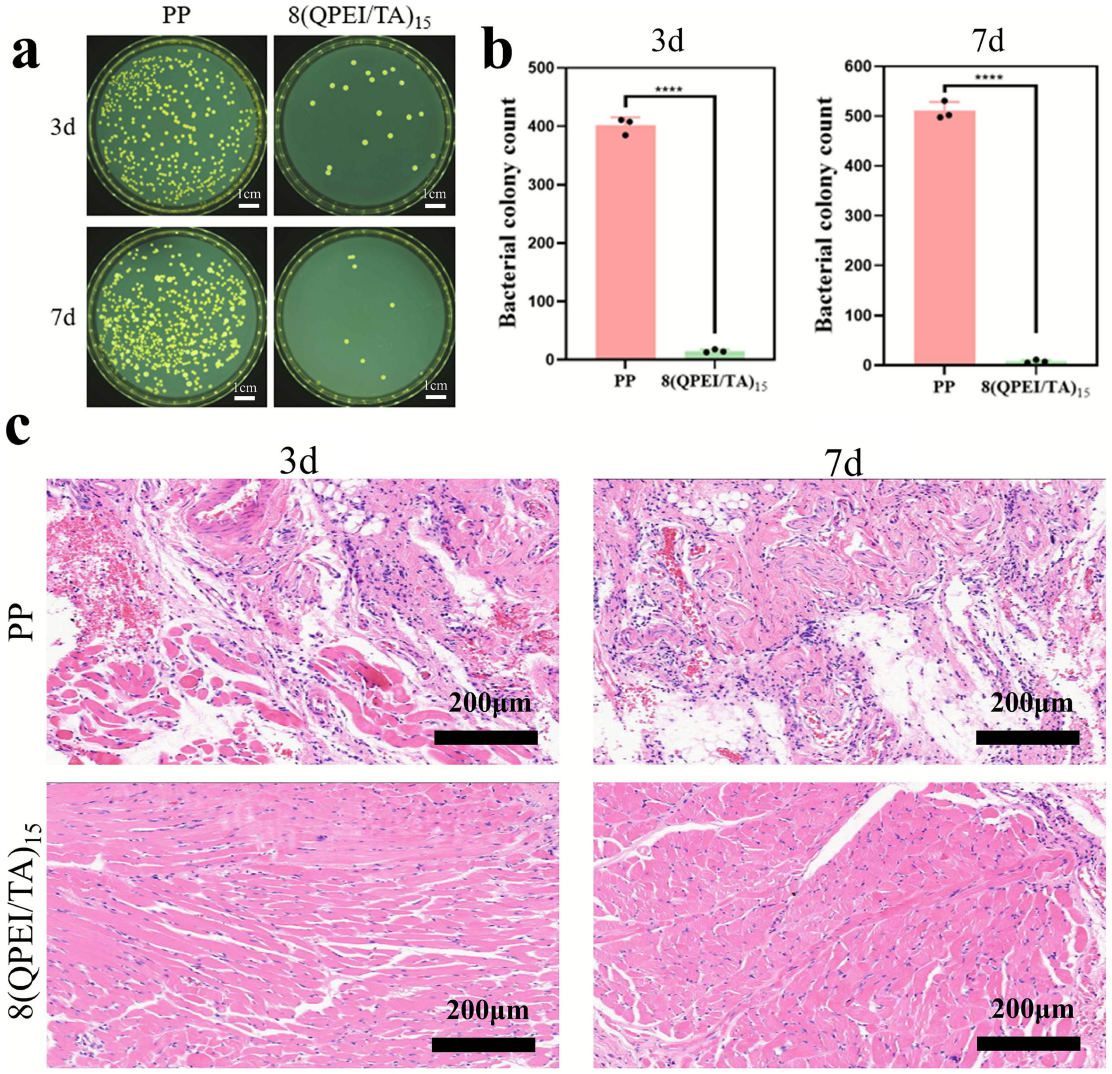
**(a)** On the 3rd and 7th day, images of viable bacteria around the tissue in contact with the material were coated on a plate, and **(b)** colony counting was performed; **(c)** H&E image of the surrounding tissue of the material (pink represents cytoplasm, bright red represents eosinophilic granules, and blue purple represents inflammatory cells) (****p < 0.0001, *n* = 3).

As shown in Figures 6a,b, the 8(QPEI/TA)_15_ group exhibited fewer bacteria compared to the PP control group, demonstrating effective bactericidal activity and reduced bacterial infection at the implantation site. Hematoxylin and eosin (H&E) staining of muscle tissue samples (Figure 6c) revealed distinct inflammatory responses between groups. At day 3 post-implantation, the PP group displayed marked acute inflammation. Substantial inflammatory cell infiltration around the tissue. This inflammatory response progressively intensified by day 7. In contrast, 8(QPEI/TA)_15_ group showed minimal inflammatory cell presence throughout the observation period. Indicating that the coating effectively eliminated *S. aureus* and prevented bacterial-induced chronic inflammation.

### 3.9 *In vivo* anti-inflammatory, antioxidant, and immune regulatory efficacy

**Figure 7:**
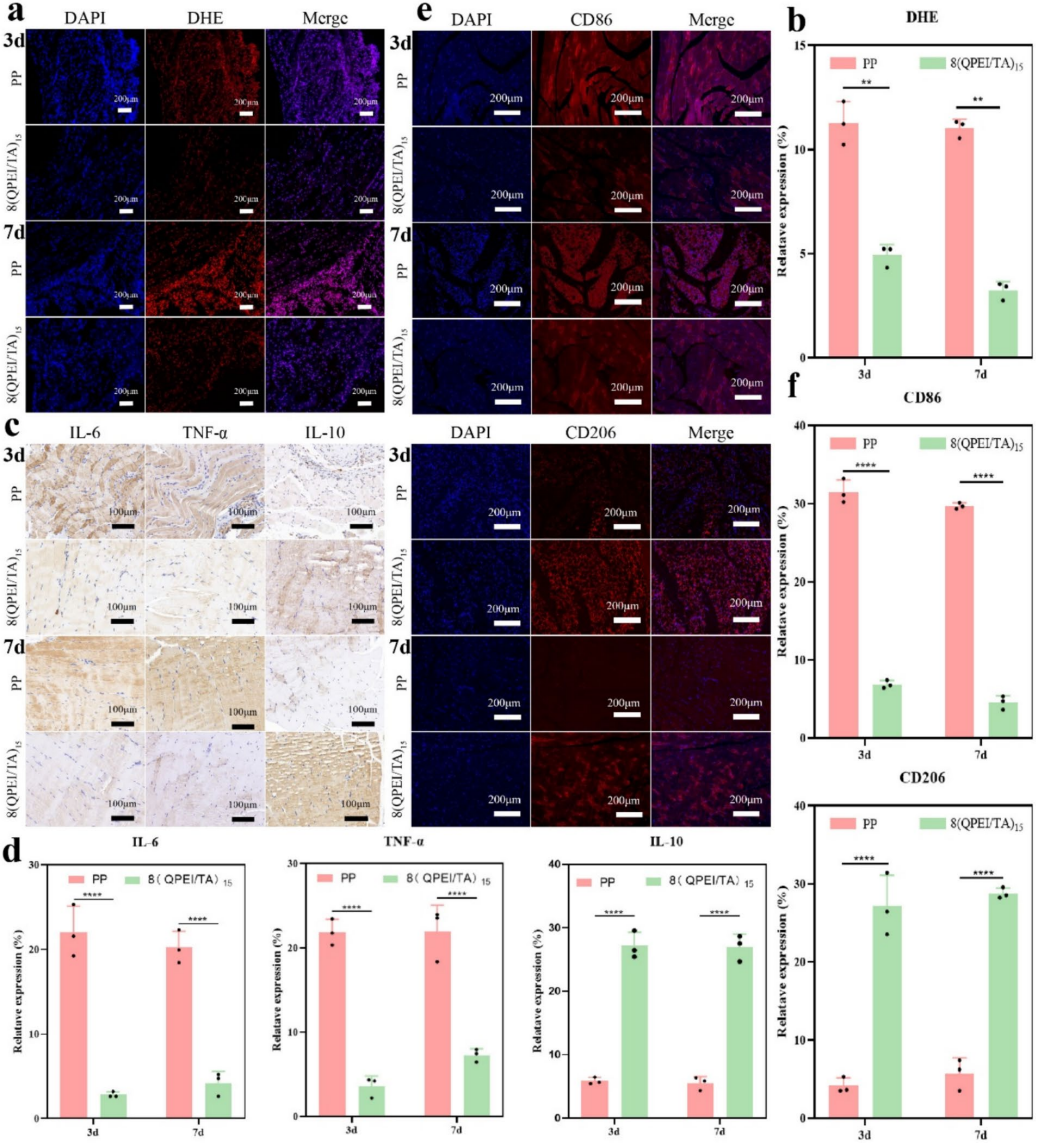
**(a)** DHE fluorescence staining images of muscle tissue surrounding the material and **(b)** DHE quantification data; immunohistochemical analysis of **(c)** TNF-α, IL-6, and IL-10 in surrounding tissues (positive results manifest as dark brown areas), and **(d)** quantitative data of TNF-α, IL-6, and TL-10; **(e)** fluorescence staining images of CD 86 and CD 206 in the surrounding tissues of the material, as well as quantitative data of CD86 and CD206 (**p < 0.01, ****p < 0.0001, *n* = 3).

**Figure 8:**
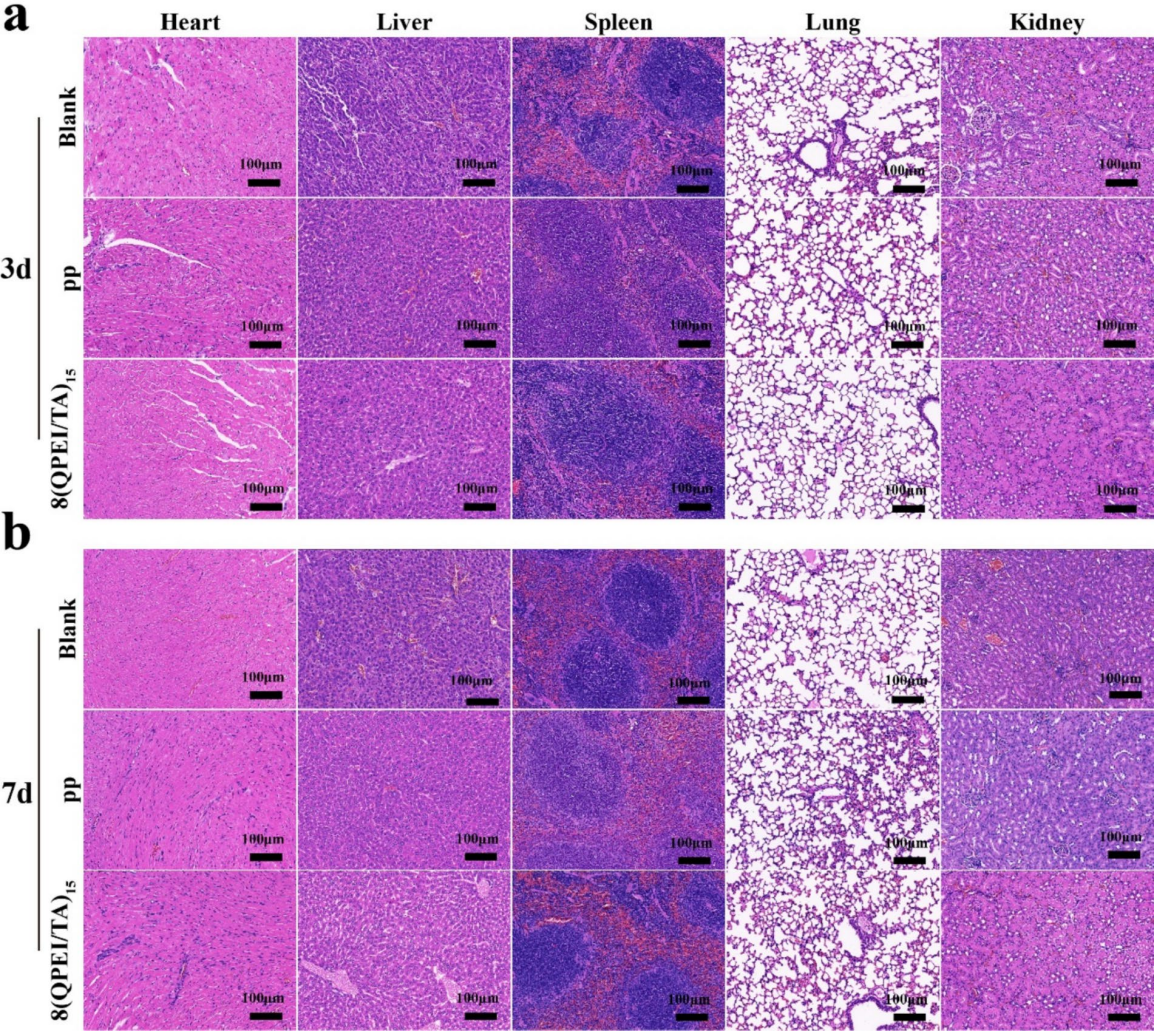
Histopathological toxicity assessment of major organs on the 3rd **(a)** and 7th **(b)** day after implantation.

On the 3rd and 7th day after implantation, DHE staining of the surrounding tissues showed intracellular ROS levels. Compared with the 8(QPEI/TA)_15_ group, the PP group showed significantly stronger DHE fluorescence intensity (Figure 7a). Quantitative analysis confirmed that the relative ROS levels of 8(QPEI/TA)_15_ group was significantly reduced, indicating their superior *in vivo* antioxidant capacity (Figure 7b). The synchronous evaluation of inflammatory cytokine expression showed significant differences among the groups. Immunohistochemical analysis of TNF-*α* and IL-6 (pro-inflammatory cytokines). Extensive positive staining in the PP group tissues on day 3. This indicating vigorous secretion of inflammatory factors. However, the expression of anti-inflammatory IL-10 remained low (Figure 7c). In contrast, the expression of TNF - α and IL-6 in group 8(QPEI/TA)_15_ was significantly reduced, while the production of IL-10 was enhanced. This anti-inflammatory effect lasted until the 7th day. Compared with the PP control group, the 8(QPEI/TA)_15_ group maintained larger IL-10 positive areas and smaller TNF-α/IL-6 positive areas (Figure 7d). The further characterization of macrophage polarization by immunofluorescence staining. At two time points, the expression of CD86 + (M1) in PP group increased and the expression of CD206 + (M2) decreased. The results of 8(QPEI/TA)_15_ group showed a decrease in CD86 + (M1) expression and an increase in CD206 + (M2) expression (Figures 7e,f). These results indicate that QPEI/TA coatings can remove reactive oxygen species (ROS). And inhibit the production of pro-inflammatory cytokines and promote the production of anti-inflammatory factors. It also can promote polarization of M2 macrophages and effectively regulate the microenvironment. Masson’s trichrome staining further demonstrated that the 8(QPEI/TA)_15_ group exhibited looser collagen fiber organization surrounding the implants compared to the PP group (Supplementary Figure S4). This finding suggests that the coating can inhibit peri-implant fibrotic tissue formation and effectively prevent tissue adhesion. At the same time, pathological toxicity assessments of the main organs were conducted on the experimental mice on the third and seventh days. The results showed that within three and seven days after implantation of the material, the main organs of the mice remained in a healthy state, and the material had no significant toxic effects on the main organs, proving that the material had good biocompatibility and safety in the *in vivo* environment (Figures 8a,b).

## 4 Conclusion

In this study, the antibacterial agent QPEI was obtained by quaternization of PEI. After screening the different groups of materials for their antibacterial properties and biocompatibility, QPEI-C6 was selected as the antibacterial agent for the surface functional layer of subsequent materials. Through layer-by-layer self-assembly technology, PP materials were modified to synthesize antibacterial materials under different concentrations and durations. Through comprehensive characterization, including material physical properties, biocompatibility assessments, ABTS and DPPH free radical scavenging assays, and antibacterial tests, the performance of the various surface functional layers was systematically evaluated. *In vivo* evaluation demonstrated that the QPEI/TA-coated material maintained significant antibacterial efficacy while exhibiting antioxidant and anti-inflammatory properties. The coating effectively regulated macrophage polarization toward the anti-inflammatory M2 phenotype, suggesting its potential for mitigating implant-associated inflammation and promoting tissue homeostasis. The results revealed that the 8(QPEI/TA)_15_ group exhibited exceptional antibacterial and antioxidant properties, along with excellent biocompatibility, demonstrating significant potential for applications in biological, medical, and related fields.

## Data Availability

The original contributions presented in the study are included in the article/Supplementary material, further inquiries can be directed to the corresponding authors.
