# Supplementary material for: A series of quaternary ammonium salt antibacterial agents synthesized and prepared for constructing and screening antibacterial coatings with biosafety on polypropylene
Source: Front Microbiol. 2026 Jan 29;17:1718331. doi: 10.3389/fmicb.2026.1718331 (PMC12894298; doi:10.3389/fmicb.2026.1718331)
Supplement: Supplementary file 1 [file Data_Sheet_1.docx]

**A Series of Quaternary Ammonium Salt Antibacterial Agents Synthesised and Prepared for Constructing and Screening Antibacterial Coatings with Biosafety on Polypropylene**

**Supplementary Methods**

QPEI with different carbon chains has been synthesized according to the above method. Characterized by Fourier transform infrared spectroscopy (FT-IR) and nuclear magnetic resonance spectroscopy. Analysis of QPEI synthesis results based on displayed characteristic peaks and chemical shifts.


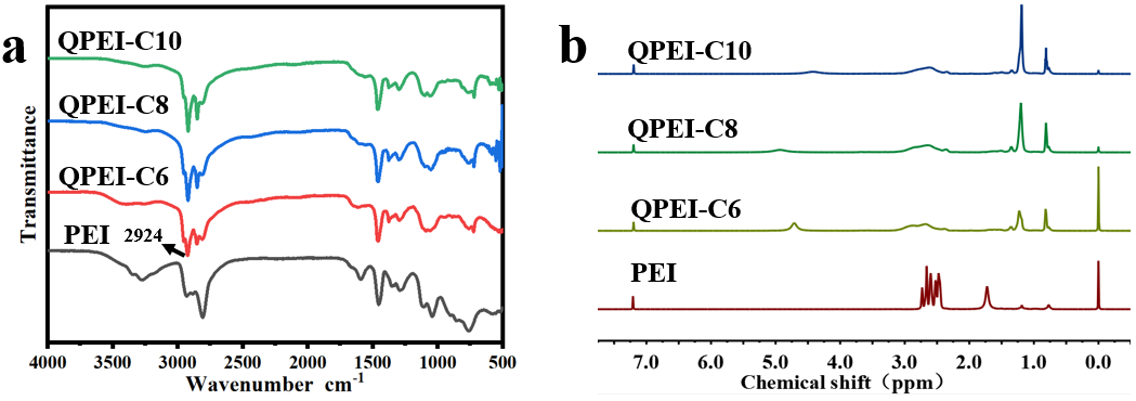


**Figure S1** FT-IR (a) and NMR (b) of QPEI with different carbon chains.


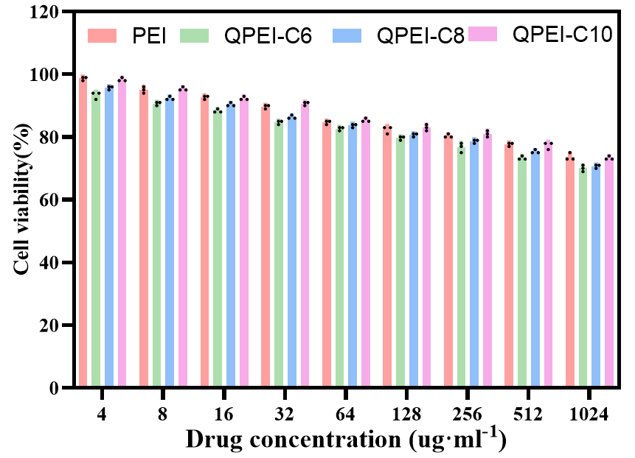


**Figure S2** Cell toxicity of three different concentrations of QPEI and PEI


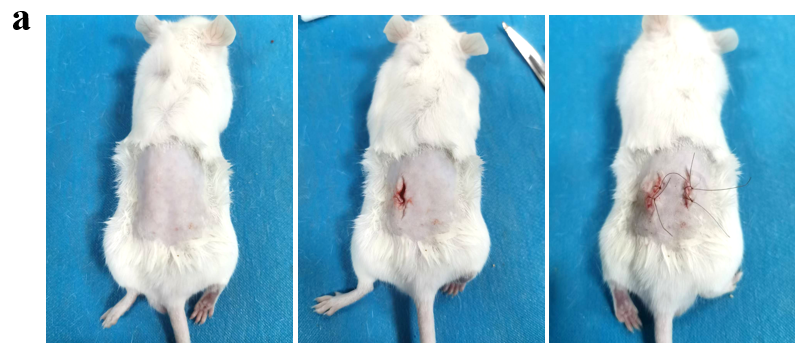


**Figure S3** (a) Photos of the animal experiment.


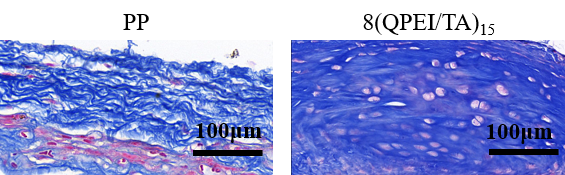


**Figure S4** (a) Masson slice staining of the surrounding tissue of the material.
